# An Alternative Approach for the Rising Challenge of Hypertensive Illness via Helicobacter pylori Eradication

**DOI:** 10.14740/cr382e

**Published:** 2015-02-09

**Authors:** Salwa A. M. Nasrat, Abdullah M. Nasrat

**Affiliations:** a Department of Physical Therapy, Cardiac Surgery Academy, Cairo, Egypt; b Department of Surgery, Balghsoon Clinic, Jeddah, KSA; c Department of Genomic Medical Research, King Fahad Research Center, KAAU, Jeddah, KSA; d Department of Surgery, KAAU, Jeddah, KSA

**Keywords:** *Helicobacter pylori*, Hypertension, Senna, Vinegar

## Abstract

**Background:**

The aim of the study was to demonstrate the effect of natural *Helicobacter pylori* eradication on blood pressure values. The prevalence of hypertension in developing countries has been considered by some reports a consequence of progress and life style changes. In spite of that, traditional risk factors do not appear fully sufficient to explain the rising figures of hypertensive illness which further indicates that attempts to control the problem depending upon traditional measures can never be adequate or decisive. *H. pylori* could migrate or get forced to migrate to the colon; it will continue producing ammonia for a reason or no reason leading to accumulation of profuse toxic amounts of ammonia, unopposed or buffered by any acidity, which could lead to multiple colonic and a high rectal spasm. A colonic re-absorptive error is established with excessive fluid and salt retention in the body that would definitely lead to hypertension which is supposed to remain inadequately controlled without correction of the underlying etiologic pathological error. It is a prospective study, conducted at Balghsoon Polyclinic, Jeddah, Saudi Arabia.

**Methods:**

Ninety-nine middle-aged male patients with essential hypertension under medications and positive for *H. pylori* dyspepsia were included in the study. They were given natural therapy for *H. pylori* eradication.

**Results:**

Ninety patients were able to resume normal blood pressure (BP) values and quit their medications.

**Conclusion:**

The concept of the colonic re-absorptive error considered in this study is not just hypothetical as upon its basis, most patients of the study (90.9%) were able to quit medications and maintain normal BP values.

## Introduction

The prevalence of hypertension continues to rise across the world, and most patients who receive medical treatment are inadequately controlled. It has been reported that tackling the global challenge of hypertension will require partnerships among multiple constituencies [1]. Hypertension, a disease of rich, is now flaring up as a challenge among poor population. Some reports consider hypertension in developing countries a consequence of progress and life style changes [2]. In spite of that, traditional risk factors do not appear fully sufficient to explain the rising figures of hypertensive illness which further indicates that attempts to control the problem depending upon traditional measures alone can never be adequate, decisive or successful.

*Helicobacter pylori* remains a challenging worldwide medical problem due to its extreme widespread prevalence, the lost quality of life of patients, the economic burden associated with its upper gastrointestinal symptoms and its close relation to acid peptic disease, gastric carcinoma and lymphoma [3-6]. About 50% of adults in the developed and 80-90% in the developing countries are estimated to be affected by *H. pylori* [7, 8]. Affection with *H. pylori* is typically life-long unless treated. It has got a clear age-related prevalence, increasing from 10% in those younger than 30 until it reaches a plateau of about 60% in those older than age of 60 or even to about 70% at 50 years of age in higher risk areas [3, 9].

Although the eradication regimens do eradicate *H. pylori* from the stomach, the emergence of antibiotic-resistant *H. pylori* strains, the severe side effects and high costs are major drawbacks of these treatments [10]. More efficient, economic and friendly drugs need to be developed.

Moreover, the flare up of a lot of challenges related to *H. pylori* through immune or different unknown reasons indicates that the current combined antibiotic therapy is not an effective measure to control all the problems caused by the stomach bug. Idiopathic hypertension and atherosclerotic stroke lie among these challenges [11-13].

Arterial hypertension is a risk factor for atherosclerosis which itself is of obscure pathogenesis; growing evidences demonstrate the causative role of endothelial dysfunction. A possible association between *H. pylori* and cardiovascular disorders has been found. The release of cytotoxic substances either of a bacterial origin or produced by the host may represent mediators of these systemic sequelae [11]. Different reports have confirmed the development and association of cytotoxin-associated gene A (cagA) positive *H. pylori* strains with many clinical problems. These reports emphasized that cagA of *H. pylori* encodes a highly immunogenic and virulence-associated protein; the presence of this virulent gene in the body could affect the clinical outcome in many patients [14].

Concerning the pathologic behavior of *H. pylori*, the organism resides and colonizes under the mucus layer overlying gastric mucosa. Although gastric acid plays an important bactericidal role, survival of *H. pylori* inside the stomach is achieved through various defense mechanisms, mainly the profuse buffering capacity of ammonia produced by the organism and the high motility of *H. pylori* even in the extremely viscid gastric mucus that also offers the organism wide range of pH gradients [6, 15-18].

*In vitro* inhibition of *H. pylori* growth was demonstrated due to the effect of some organic acids, lactic, formic and acetic, with the lactic acid demonstrating the greatest inhibition due to the effect of feed back regulation and product inhibition as the main product of glucose utilization by *H. pylori* is recognized as lactate [19].

This study aimed to demonstrate the effect of natural *H. pylori* eradication from the colon on blood pressure (BP) values in patients with hypertension under medication associated with *H. pylori* dyspepsia.

## Patients and Methods

A prospective study was held in Balghsoon Polyclinic, Saudi Arabia during the period from May 2011 to October 2013. Hypertensive patients under medication with frank history of *H. pylori* dyspepsia were randomly included in this study without any selection except smokers and those with an associated chronic illness like diabetes who were excluded. The study included 99 different nationality male patients living in Saudi Arabia. The age of patients ranged between 25 and 55 years and they were receiving different groups of antihypertensive medications. Their body weight ranged between average to well-built; seven patients (7.07%) were slim and three patients (3.03%) were overweight. The range of their systolic BP under medication was 140 - 155 mm Hg, and the diastolic range was 90 - 105 mm Hg. The following *H. pylori*-related dyspeptic symptoms were considered, hyperacidity, stomach upsets, acid reflux, indigestion, abdominal distension after meals and constipation. The lipid profile for all patients had been within normal range without any medical treatment. Their hypertensive illness was essential and was not secondary to any organic disorder.

*H. pylori* serum antibody test, though non-specific, was used for screening of all patients as being cost effective. *H. pylori* existence was confirmed in all patients by reliable specific tests, urea breath test and *H. pylori* fecal antigen [20]. Routine colonoscopy was done for all patients in order to exclude colonic pathology. Patients demonstrating significant colonic pathology such as diffuse ulcerations or multiple polyposis were preferably excluded as they would not tolerate or feel easy with the natural remedies used in the study.

The *H. pylori* serum antibodies test was available from Semen Co., USA with Batch No. 104132 while the urea breath test was available from Helicap Co., Sweden with Batch No. HCO1150108-E10. The *H. pylori* fecal antigen test was obtained from Acon Laboratory, USA, Batch No. HP8040008.

A colon clear by the natural senna leaves purge was employed for all patients as a primary step in order to eradicate the migrated colonic *H. pylori* strains [21]. All patients followed a natural remedy for gastric and colon care to complete eradication of abnormal behavior *H. pylori* strains; 20 cc of dietary white vinegar 5-6% mixed with a food staff (white cheese, mashed potato or yoghurt which is the best preferred food stuff for the vinegar to be mixed with), two times daily during meals for 7 - 10 days [21]. Confirmation of *H. pylori* eradication by urea breath test and *H. pylori* fecal antigen was done at end of natural therapy [20]. All individuals included in the study were treated as outpatients.

### Ethical considerations

An informed signed consent was taken from all patients, and they were free to quit the study whenever they like. All patients were allowed to follow their usual diet, medications and to lead their routine life style. The research proposal was approved and the study followed the rules of the Research Ethics Committee of King Abdul-Aziz University (KAAU) in Jeddah, Saudi Arabia.

## Results

Dyspepsia constituted a major disturbance to the quality of life for all patients. Severe constipation manifested by passage of small pieces of dried stool together with pitting edema opposite the shaft of the leg were constant features in all patients. All patients were positive for *H. pylori* serum antibodies, while urea breath test and *H. pylori* fecal antigen confirmed this diagnosis. A high rectal spasm was demonstrated by proctoscopy or sigmoidoscopy in all patients while colonoscopy showed multiple colonic spasms in most patients. None of them demonstrated any further significant colonic pathology.

Data from pre-treatment observational findings showed that patients were having better BP readings whenever they had regular comfortable bowel motions, while their BP was badly controlled when they suffer from indigestive troubles or abdominal distension.

All patients expressed dramatic relief concerning their dyspeptic symptoms and constipation was relieved in all patients after 3 - 5 days of treatment. All patients were confirmed negative for *H. pylori* after the natural therapy.

Fifty-seven patients (57.6%) were able to quit their medications and maintain normal BP values after the vinegar therapy. Thirty-three patients (33.3%) needed an additional 1 week of therapy with vinegar in order to resume a normal BP and quit medication. Six patients (6.06%) failed to get their BP lowered after therapy with both the vinegar and the purge; the three with overweight were not among them. Their failure to respond to therapy was attributed possibly to gaining new *H. pylori* strains due to misbehavior in food habits via outside-home meals. Three patients (3.03%) did not complete the study.

Patients who responded to the natural therapy were followed up after 1 month, 3 months, 6 months, 1 year and 18 months. Their BP remained controlled within normal levels without any medications. They had been made aware that they should watch their outside-home meals and their condition of the colon as they might need to return to the natural purge and vinegar in their food whenever they develop any dyspeptic symptoms or whenever they feel upset after any query meal.

An equal group of patients of an equal size, similar age range, body built, range of BP, associated *H. pylori* dyspepsia and nearly most of other circumstances as not having nor receiving any medication for any other chronic illness was considered as control and was followed up for the same duration. They were not interested in the natural therapy with senna purge and vinegar and they decided to follow medical treatment with antihypertensive pills. They mostly remained inadequately controlled in spite of regular follow-up of medications and carefulness about their life style.

## Discussion

Hypertension is considered a major health problem worldwide; it is one of the leading causes of death and disability in many countries. The public health response towards this ongoing pandemic challenge must be promoted and the policies of health organizations need to be re-oriented to include this chronic disease within their prior attention [1, 2].

Functional dyspepsia is a clinical syndrome defined by chronic or recurrent pain or discomfort in the upper abdomen of a variable origin. A general agreement exists on the irrelevant role played by *H. pylori* in the pathophysiology of functional dyspepsia. *H. pylori* represents one of the most common and prominent topics worldwide; it is becoming exceedingly a challenging medical problem [3].

A remarkable association between *H. pylori* and cardiovascular disease was documented in literature and it was suggested to be due to a higher prevalence of more virulent *H. pylori* strains. It has been reported that *H. pylori* eradication improves BP values in patients affected by hypertension. The link between hypertensive disease and *H. pylori* was related to a possible activation of the cytokine cascade with the release of vasoactive substances from the primary site of *H. pylori* colonization [11-13].

Concerning the clinical picture of *H. pylori*, acute conditions include upper gastrointestinal pain, burping, gastric distension, halitosis, hyperacidity and later hypochlohydria, while chronic cases could be asymptomatic. Gastric acid secretion is stimulated during early stages by the inflammatory process and by the juxtamucosal ammonia produced by the organism, while hypochlorhydria develops later due to mucosal atrophy [6, 16]. It seems that the medical problems related to *H. pylori* creep up under the silence of chronic existence of the bacterium.

The controversy about the efficacy of *H. pylori*-antibiotic eradication strategies and the emergence of antibiotic-resistant *H. pylori* strains have been illustrated in literature [10, 20]; the apparent length of antibiotic therapy per se could be the reason to allow the chance for the stomach bug to develop resistance or to escape from the stomach. Antibiotics are seldom effective in patients harboring extra-gastric *H. pylori* strains [22]. *H. pylori* could migrate to the colon [20], or the antibiotic violence itself does force it to migrate where antibiotics will become ineffective against it.

Migration of *H. pylori* to the colon is a fact that has been documented in literature and colonic colonization with these abnormal-behavior *H. pylori* strains would be life-long unless eradicated [9, 10, 20]. The organism will continue producing ammonia for a reason or no reason with consequent accumulation of profuse toxic amounts of ammonia in the colon, un-opposed or buffered by any acidity. As ammonia in residual amounts is smooth muscle tonic and smooth muscle spastic in profuse amounts, its accumulation in the colon would lead to development of multiple colonic and a high rectal spasm. A colonic re-absorptive error could be established with excessive fluid and salt retention in the body that would definitely lead to hypertension which is supposed to remain inadequately controlled without correction of the underlying etiologic pathological error [21]. The passage of small pieces of dry stool as expressed by the patients and the pitting edema demonstrated over their legs were explained by the development of colonic spasms and the colonic re-absorptive error with fluid and salt retention in the body. In this study, the high rectal spasm was demonstrated by proctoscopy or sigmoidoscopy while the colonic spasms were confirmed by colonoscopy.

It has been demonstrated in literature findings that the incidence of *H. pylori*-associated medical problems is higher in developing countries than in developed ones due to the higher prevalence of *H. pylori* in developing communities [20], possibly because of the hygienic and life standards and/or the abuse of antibiotics. The subject of *H. pylori* is a sanitary conflict before it is a medical challenge [8, 23-25]. The patients included in this study were from different countries; there was no exclusion of patients because of nationality so long they were living sufficient years in the same community, as the matter of the sanitary conflict is food habits and community-related rather than racial or nationality-related.

Observational findings in this study have shown that the body weight of patients has got insignificant influence on the degree of their *H. pylori*-associated hypertension; therefore, the body mass indices of patients were not taken into detailed consideration or discussion in the study. The matter is apparently dependant mainly upon the degree of fluid and salt retention in the body which should be related to the rate of colonization of abnormal behavior *H. pylori* strains in the colon rather than the body weight of the patient.

The follow-up of patients was rather long as those patients are susceptible disadvantaged population liable for recurrence whenever they develop colonic troubles; therefore, they were instructed to care for their colon and watch their meals from outside home.

Dietary vinegar has been recently demonstrated as an effective natural remedy against *H. pylori* including symptomatic relief and eradication [19, 21]. The complex nutritional requirements of *H. pylori* are achieved through its unique energy metabolism, which exhibits characteristic dislocation sites. These sites can be considered as targets that should attract any attempts to fight the organism [10, 26]. The major routes of generation of energy of *H. pylori* are via pyruvate and the activity of the pyruvate dehydrogenase complex is controlled by the rules of product inhibition and feedback regulation. As acetate is demonstrated as an end product among the metabolic pathway of *H. pylori*, this means that addition of acetic acid to the atmosphere around the organism could compromise the energy metabolism of *H. pylori* or interfere with the organism’s respiratory chain metabolism [27-30]. This fact is further supported by the observation that addition of pyruvate to different solid culture media was found to inhibit bacterial growth, and this inhibition was attributed to accumulation of acetate and formate [19, 27]. As the matter includes interference with the energy metabolism and the respiratory chain of *H. pylori*, an immediate paralysis of the organism can be considered which further explains the dramatic symptomatic relief that has been mostly expressed by all patients taking a vinegar-mixed food. The fast immediate influence of acetic acid on *H. pylori* gives no chance for the organism to resist the treatment with vinegar and migrate or develop resistant strains.

*In vitro* inhibition of *H. pylori* growth was demonstrated due to the effect of some bio-organic acids, lactic, formic and acetic with the lactic acid demonstrating the greatest inhibition as the main product of glucose utilization by *H. pylori* is recognized as lactate [19]. Therefore, yoghurt was chosen in this study as the best food stuff for the vinegar to be mixed with, as it will assist the inhibitory effect of vinegar on *H. pylori* according to the rules of feed back regulation and product inhibition.

In this study, the effect of pyruvate, 20 times dilutions of dietary vinegar (acetic acid 6%) and three times dilution of the senna purge preparation added to *H. pylori* solid culture media was studied. Addition of pyruvate demonstrated a delayed inhibitory effect on the motility of *H. pylori*, while addition of the diluted vinegar and the diluted senna leaves extract showed an immediate lethal influence on *H. pylori*.

### Conclusion

The concept of the colonic re-absorptive error considered in this study is not just hypothetical as upon the basis of this concept; most of the patients of the study (90.9%) were able to quit their medications and maintain normal BP values by mere natural eradication of *H. pylori*, colon care and colon clear, although they were inadequately controlled in spite of regular follow-up of medications and extreme carefulness about their style of life.

Revision of the current guidelines of *H. pylori* eradication and management of hypertension may be needed. Therapy with dietary vinegar and natural senna purge is a promising remedy and is worthy of further accurate determination and wider practical applications.
